# Recent Progress in Additive Manufacturing of Alloys and Composites

**DOI:** 10.3390/ma17122905

**Published:** 2024-06-13

**Authors:** Feiyang Gao, Haizhou Lu, Chao Zhao, Haokai Dong, Lehua Liu

**Affiliations:** 1 National Engineering Research Center of Near-Net-Shape Forming for Metallic Materials, Guangdong Provincial Key Laboratory for Processing and Forming of Advanced Metallic Materials, South China University of Technology, Guangzhou 510640, China; gfy20018@163.com (F.G.); donghk@scut.edu.cn (H.D.); 2 School of Mechatronic Engineering, Guangdong Polytechnic Normal University, Guangzhou 510665, China; hzlu@gpnu.edu.cn; 3 School of Materials Science and Engineering, Huazhong University of Science and Technology, Wuhan 430074, China; czhao@hust.edu.cn

## 1. Introduction

Additive manufacturing, commonly referred to as 3D printing, is a fabrication method characterized by a layer-by-layer deposition process [1]. Its procedural framework encompasses several sequential stages, including design conceptualization, computer-aided modeling, slicing of digital designs into printable layers, physical printing, and subsequent post-processing treatments. In contrast to conventional manufacturing methodologies, additive manufacturing presents notable advantages such as enhanced geometric versatility, reduced material wastage, and expedited production cycles [2]. Furthermore, the distinctive attributes of additively manufactured alloys and composites, which include a profusion of metastable microstructures and exceptional material properties, have attracted considerable scholarly and industrial attention. These attributes are primarily ascribed to the inherent characteristics of additive manufacturing processes, including rapid cooling rates, large thermal gradients, and intricate thermal cycling histories. As a result, additive manufacturing has emerged as a focal point of interdisciplinary research endeavors, garnering widespread interest and engagement from academic and industrial stakeholders alike.

Currently, additive manufacturing is extensively applied in the fabrication of steels [3], nonferrous alloys [4], and metal matrix composites [5], meeting the stringent performance requirements across diverse sectors including aerospace, automotive, electronics, medical, military, and architecture. However, the additive manufacturing of alloys and composite materials still faces significant challenges. These challenges encompass an inadequate understanding of the complex interactions between processing parameters, the resulting microstructures, and the ensuing material properties. Additionally, elevated production costs, the prevalence of defects, and a lack of established theories elucidating the principles of physical metallurgy governing additive manufacturing processes further complicate the advancement and optimization of these technologies.

To further advance additive manufacturing technology, Dr. Liu et al. recently curated a Special Issue entitled “Additive Manufacturing of Alloys and Composites”. This Special Issue focuses on advanced materials and related processes, aiming to explore the mechanisms governing microstructural evolution and property enhancement in additive manufacturing. Ultimately, ten contributions showcasing significant advancements in this domain were selected for inclusion. These articles cover additively manufactured stainless steels, superalloys, CoCrMo alloys, and metal matrix composites. Through these publications, this Special Issue not only presents the latest developments in high-performance additive manufacturing materials but also paves the way for future advancements in additive manufacturing technology and the development of advanced materials.

## 2. Contributions

### 2.1. Alloys

Stainless steel, with its excellent mechanical properties and corrosion resistance, is gaining increasing attention in both scientific and technological fields. Chang et al. [6] successfully prepared complex 15-5PH stainless steel components by combining powder metallurgy with fused deposition modeling (FDM). Their results demonstrated optimal material fluidity at 285 °C during the FDM process and a solvent debinding rate of 98.7% achieved at 75 °C over 24 h. Furthermore, during the sintering process, the relative density of the sintered parts reached 95.83% at a sintering temperature of 1390 °C. This study provides valuable guidance for optimizing the FDM, debinding, and sintering processes.

In contemporary industrial applications, duplex stainless steels (DSSs) are extensively utilized across various sectors. However, the challenges in producing additive-manufactured DSSs are compounded by high levels of nitrogen (N), chromium (Cr), molybdenum (Mo), and other alloying elements. To address these issues, He et al. [7] developed a novel low-N 25Cr-type DSS. This novel low-N 25Cr-type DSS, fabricated using the Laser Powder Bed Fusion (L-PBF) method, exhibited a yield strength of 712 MPa and an elongation of 27.5%. Moreover, this study revealed that solution treatment at 1200 °C leads to the formation of discrete and refined austenite precipitates at ferrite grain boundaries, thereby enhancing strength and ductility. In their subsequent work [8], they transferred their eyes to the corrosion resistance investigation of low-N 25Cr-type duplex stainless steel prepared by the L-PBF method and solution treatment and analyzed the mechanism behind corrosion resistance enhancement. The results showed after solution treatment at 1200 °C for 1 h, the residual thermal stress in the specimen was eliminated and the Cr content in the ferrite phase increased, leading to an improvement in corrosion resistance.

To mitigate the stress shielding phenomenon in bone implants, Lam et al. [9] employed a selective laser melting (SLM) process to fabricate porous CoCrMo alloys. Their study aimed to determine the optimal volume porosities and heat treatment parameters to achieve a close match in elastic modulus and yield strength between human cortical bone and SLM-built CoCrMo alloys. Their results revealed a significant reduction in elastic modulus and yield strength with increasing actual porosity. Notably, heat-treated CoCrMo structures with an actual porosity of 48% demonstrated the most favorable mechanical properties, closely approximating those of human cortical bone, thereby presenting promising potential for biomedical implant applications.

Additionally, Jiang et al. [10] outlined an optimal process route for the preparation of Inconel 718 tools intended for cold, deep drawing applications. Utilizing Inconel 718 powder as the raw material, these authors optimized parameters for both Laser Powder Bed Fusion (L-PBF) and double annealing (DA), followed by surface finishing techniques. The resulting Inconel 718 tools exhibited a tensile strength of 1511.9 MPa and a hardness of 55 HRC. This process route successfully addressed the challenge of meeting the mechanical property requirements for cold deep drawing applications with L-PBF-built Inconel 718 tools.

### 2.2. Metal Matrix Composites

The thermal conductivity of metal matrix composites is a critical property for die production and electronic devices. Determining the effective thermal conductivity of additive-manufactured composites under various parameters often necessitates extensive experiments. Li et al. [11] addressed this challenge by constructing a theoretical model with a high accuracy of 86.7% for rapidly predicting the thermodynamic properties of laser-cladded Cu/Ni composites. The model’s error was attributed to the ignorance of metallurgical bonding at the interface between the two different metals during the laser cladding process. It was observed that higher model accuracy could be achieved in samples with a larger cladding layer thickness. Meanwhile, Zhou et al. [12] employed their independently designed liquid–solid separation method to fabricate diamond/Al composites reinforced with 40 vol.% diamond particles, achieving high thermal conductivity. This innovative approach demonstrates the potential for producing composites with superior thermal properties for advanced industrial applications.

To ensure excellent interfacial bonding in Fe/Al composites post-heat treatment, a comprehensive understanding of the growth kinetics of intermetallic compounds (IMCs) is crucial. Zhang et al. [13] investigated the nucleation and early growth behavior of pure Fe/pure Al IMCs using in situ analysis. During the heat treatment process at 380 °C, the primary IMCs transitioned from the initial Fe_4_Al_13_ phase to the stable Fe_2_Al_5_ phase. Initially, the growth rate of IMCs in thickness was closely aligned with the horizontal growth rate, ranging from 0.02 to 0.17 µm/min. However, upon reaching a thickness of 4.5 µm, the growth rate significantly decelerated to 0.007 µm/min. Similarly, during heat treatment at 520 °C, the predominant IMCs were of the Fe_2_Al_5_ phase, exhibiting a horizontal growth rate of 0.53 µm/min and a thickness growth rate of 0.23 µm/min. This investigation into the nucleation and early growth behavior of IMCs provides valuable insights, potentially reducing the reliance on costly trial-and-error processes in the microalloying of these composites.

The aerospace and transportation industries impose stringent requirements on the mechanical and damping properties of Al-based matrix composites (AMCs). Insufficient damping capacity has notably restricted their applications in vibration-sensitive environments. Lin et al. [14] investigated the mechanical and damping properties of SiC_f_/Al-Mg composites with varying Mg contents. These composites were fabricated using colloidal dispersion combined with a squeeze-melt infiltration process. Their results indicated that the addition of Mg effectively enhanced interface bonding and strengthened the aluminum matrix, thereby improving the flexural strength and elastic modulus of the composite. However, higher Mg content led to the formation of pores, compromising the composite’s plasticity. Furthermore, the incorporation of SiC fibers significantly enhanced the damping capacity of the composites, particularly under strain amplitudes exceeding 0.001%. This study provides valuable insights into achieving AMCs with superior mechanical performance.

As high-speed and heavy-haul rail transportation continue to evolve, there is an increasing demand for materials with a superior combination of hardness and toughness to prevent surface failures in rail turnouts. Zhao et al. [15] developed in situ WC primary reinforced bainite steel matrix composites with a notable hardness/toughness trade-off using direct laser deposition (DLD). These authors attributed the excellent balance between hardness and toughness to the adaptive adjustment of both the matrix and reinforcement microstructures, where a higher concentration of primary reinforcement contributed to enhanced mechanical properties.

## 3. Outlook

The present Special Issue attracted a substantial number of submissions, from which over 10 exceptional works were meticulously chosen to comprise the final publication. The Guest Editors express their sincere gratitude to all authors, reviewers, and publishers for their contributions and support of this endeavor. Encouraged by this success, a new Special Issue entitled “Additive Manufacturing of Alloys and Composites (Second Edition)” has been commissioned. Submissions from researchers worldwide are welcome for consideration in this forthcoming Special Issue, with further details available on the following website: https://www.mdpi.com/journal/materials/special_issues/NB0LNF40YT (accessed on 20 February 2024).
